# Neurophysiological explorations across the spectrum of psychosis, autism, and depression, during wakefulness and sleep: protocol of a prospective case–control transdiagnostic multimodal study (DEMETER)

**DOI:** 10.1186/s12888-023-05347-x

**Published:** 2023-11-21

**Authors:** Valeria Lucarini, Anaëlle Alouit, Delphine Yeh, Jeanne Le Coq, Romane Savatte, Mylène Charre, Cécile Louveau, Meryem Benlaifa Houamri, Sylvain Penaud, Alexandre Gaston-Bellegarde, Stéphane Rio, Laurent Drouet, Maxime Elbaz, Jean Becchio, Sylvain Pourchet, Estelle Pruvost-Robieux, Angela Marchi, Mylène Moyal, Aline Lefebvre, Boris Chaumette, Martine Grice, Påvel G. Lindberg, Lucile Dupin, Pascale Piolino, Cédric Lemogne, Damien Léger, Martine Gavaret, Marie-Odile Krebs, Anton Iftimovici

**Affiliations:** 1 Université Paris Cité, Institute of Psychiatry and Neuroscience of Paris (IPNP), INSERM U1266, Team “Pathophysiology of psychiatric disorders”, GDR 3557-Institut de Psychiatrie, 102-108 Rue de la Santé, Paris, 75014 France; 2 GHU Paris Psychiatrie et Neurosciences, Pôle Hospitalo-Universitaire d’évaluation, Prévention, et Innovation Thérapeutique (PEPIT), Paris, France; 3 Université Paris Cité, Institute of Psychiatry and Neuroscience of Paris (IPNP), INSERM U1266, Team “Stroke: from prognostic determinants and translational research to personalized interventions”, Paris, 75014 France; 4 Laboratoire Mémoire, Cerveau et Cognition, UR7536, Université Paris Cité, Boulogne-Billancourt, F-92100 France; 5 Centre du Sommeil et de la Vigilance, AP-HP, Hôtel-Dieu, Paris, France; 6 Collège International de Thérapies d’orientation de l’Attention et de la Conscience (CITAC), Paris, France; 7 Service de Neurophysiologie Clinique, GHU Paris Psychiatrie et Neurosciences, Paris, France; 8 Epileptology and Cerebral Rhythmology, APHM, Timone Hospital, Marseille, France; 9 Department of Child and Adolescent Psychiatry, Fondation Vallee, UNIACT Neurospin CEA - INSERM UMR 1129, Universite Paris Saclay, Gentilly, France; 10 IfL-Phonetics, University of Cologne, Cologne, Germany; 11 INCC UMR 8002, CNRS, Université Paris Cité, Paris, F-75006 France; 12 Inserm, INRAE, Center for Research in Epidemiology and StatisticS (CRESS), Service de Psychiatrie de l’adulte, AP-HP, Hôpital Hôtel-Dieu, Université Paris Cité and Université Sorbonne Paris Nord, Paris, France; 13 VIFASOM, ERC 7330, Université Paris Cité, Paris, France

**Keywords:** Neurophysiology, EEG microstates, Sleep, Sensorimotor integration, Speech, Virtual reality, Psychosis, Autism, Depression, Sense of self

## Abstract

**Background:**

Quantitative electroencephalography (EEG) analysis offers the opportunity to study high-level cognitive processes across psychiatric disorders. In particular, EEG microstates translate the temporal dynamics of neuronal networks throughout the brain. Their alteration may reflect transdiagnostic anomalies in neurophysiological functions that are impaired in mood, psychosis, and autism spectrum disorders, such as sensorimotor integration, speech, sleep, and sense of self. The main questions this study aims to answer are as follows: 1) Are EEG microstate anomalies associated with clinical and functional prognosis, both in resting conditions and during sleep, across psychiatric disorders? 2) Are EEG microstate anomalies associated with differences in sensorimotor integration, speech, sense of self, and sleep? 3) Can the dynamic of EEG microstates be modulated by a non-drug intervention such as light hypnosis?

**Methods:**

This prospective cohort will include a population of adolescents and young adults, aged 15 to 30 years old, with ultra-high-risk of psychosis (UHR), first-episode psychosis (FEP), schizophrenia (SCZ), autism spectrum disorder (ASD), and major depressive disorder (MDD), as well as healthy controls (CTRL) (*N* = 21 × 6), who will be assessed at baseline and after one year of follow-up. Participants will undergo deep phenotyping based on psychopathology, neuropsychological assessments, 64-channel EEG recordings, and biological sampling at the two timepoints. At baseline, the EEG recording will also be coupled to a sensorimotor task and a recording of the characteristics of their speech (prosody and turn-taking), a one-night polysomnography, a self-reference effect task in virtual reality (only in UHR, FEP, and CTRL). An interventional ancillary study will involve only healthy controls, in order to assess whether light hypnosis can modify the EEG microstate architecture in a direction opposite to what is seen in disease.

**Discussion:**

This transdiagnostic longitudinal case–control study will provide a multimodal neurophysiological assessment of clinical dimensions (sensorimotor integration, speech, sleep, and sense of self) that are disrupted across mood, psychosis, and autism spectrum disorders. It will further test the relevance of EEG microstates as dimensional functional biomarkers.

**Trial registration:**

ClinicalTrials.gov Identifier NCT06045897.

## Background

In light of the genetic and neuroanatomical continuum among psychiatric illnesses [1, 2], transdiagnostic neurophysiological approaches have demonstrated shared neurofunctional abnormalities between schizophrenia, mood, and autism spectrum disorders [3, 4]. High-level cognitive processes have long been shown to rely on synchronized neuronal oscillations, resulting from a balance between excitatory and inhibitory populations of neurons, which can be directly measured by electroencephalography (EEG). This balance is maintained by a network of GABAergic interneurons that regulate the activity of superficial pyramidal cells [5]. The topographically precise inhibitory activity of interneurons also allows for spatial sensory coding, which is key to a variety of memory processes [6]. Disruption in these systems may therefore explain a range of cognitive symptoms seen across the spectrum of psychiatric disorders. Moreover, the timing of the disruption may explain the neurodevelopmental continuum between autism spectrum on the one hand, and psychotic and mood disorders on the other. For instance, glutamatergic NMDA receptors, which regulate interneuron activity, can be affected by genetic mutations disrupting the function of subunits expressed either in early development or later on, leading respectively to neurodevelopmental phenotypes, such as ASD, or to schizophrenia spectrum-disorders [7]. In addition, interneurons are crucial to prefrontal maturation during adolescence and early adulthood [8], a timeframe when most psychiatric disorders occur [9]. EEG quantitative approaches therefore appear as accessible and promising tools to investigate the pathophysiology of psychiatric disorders, from autism to schizophrenia spectrum disorders.

Beyond frequential or oscillatory activities, EEG analyses now allow to study the temporal dynamics of neuronal networks throughout the brain [10]. At rest, brain activities alternate very rapidly, every 80 ms or so, between states of unstable equilibrium, called microstates, and characterized by a particular polarization of the entire cerebral electrical potential field. EEG coupled with functional MRI has suggested that these microstates may correspond to particular modes of spatial organization of information processing [11]. For instance, microstate classes have been associated with various functioning profiles: verbal (class A), visual (class B), self-oriented/self-referential processing (class C), cognition (class D), and interoception and sensorimotor processing (class E) [12]. Moreover the same microstate structures have been described from waking rest to deep sleep, confirming that they may reflect a robust large-scale resting-state network architecture, similar to the resting-state connectivity seen in fMRI that is also preserved in sleep [13]. Disruption in these microstate systems has been described across the spectrum of psychiatric disorders, in schizophrenia [14], autism spectrum disorder [15], or depression [16], but also in neurological disorders such as epilepsy [17]. Thus, preliminary results from our group suggested that a certain pattern of microstates could be associated with specific stages of disease progression in psychosis, but also translated a shared dimension on the schizophrenia-autism continuum [18]. They may therefore contribute to understanding the range of transdiagnostic endophenotypes shared between neuropsychiatric diseases, such as anomalies in sleep, sensorimotor integration, speech, and sense of self. Moreover, since microstates can be modulated under hypnotic conditions [19], and medical hypnosis has been associated with improved attentional and executive control over self-referential processes [20], EEG microstates may also provide a proxy for psychotherapeutic response. Thus, the effectiveness of hypnosis has been suggested in psychiatric disorders associated with overactivation of the default mode, such as depression [21].

### Sleep

Sleep disturbances are strongly linked to the pathophysiology of most neuropsychiatric disorders and may explain many of the cardiovascular, pneumologic, and neurologic comorbidities of psychiatric disorders [22]. Various mechanistic models have been described in bipolar disorder and depression, including circadian rhythm anomalies, internal desynchronization, or anomalies of sleep architecture [23, 24]. Sleep dysregulation is also highly prevalent in autism spectrum disorders, leading to severe distress and impact on quality of life [25], while in the early stages of psychosis, there is a high prevalence of insomnia, nightmare disorder, sleep-related hallucinations, excessive sleepiness disorders or restless leg syndromes [26]. Moreover, having a sleep disorder exacerbates psychotic and mood symptoms among patients with psychosis [26]. From a neurophysiological perspective, the most replicated macroscopic EEG anomaly across the spectrum of psychosis, mood disorders, and autism, is the decrease in density of sleep spindles [27–29], which are determinant for cognitive processes such as memory consolidation [30]. However, more quantitative EEG analyses remain to be done to further explore brain connectivity during sleep.

### Sensorimotor integration

Sensorimotor abnormalities are a cross-cutting neuropsychiatric dimension [31–33], which can be robustly analyzed with quantitative EEG [34]. Motor deficits linked to alterations in cortical excitability/inhibition modulation of motor areas have been identified in various neurodevelopmental pathologies such as schizophrenia or autism [35, 36], and in particular during adaptation to a probabilistic context [37].

Specifically, autism spectrum disorders have been shown to exhibit anormal context-sensitive processing mechanisms, sensorimotor gating deficits, as well as repetitive motor movements and atypical integration of sensory stimuli [38–40]. Recent behavioral and imaging studies investigating tactile processing in autism, suggested no difference in light touch detection and texture, but increased sensitivity in vibration [41–43]. Moreover, decreased connectivity in finger somatosensory areas and slower perceptual processing speed were shown [44, 45]. Although sensory perturbations are well known, literature on sensory integration prior to motor movement is lacking. In schizophrenia, and more generally in psychotic disorders, it is still unclear how sensorimotor mechanisms are impaired. It has been hypothesized that a general disruption may cause a functional disintegration between sensory and cognitive processes [32, 46], yet, further investigations are needed in order to shed light on precise sensorimotor integration. Only a handful of studies showed tactile perception accuracy deficits [47–49], and abnormal sensory predictions in a self- and non-self-elicited sensation discrimination task [50]. This indicates a failure of normal inhibitory regulation of sensory, motor, and attentional mechanisms, common in several neurodevelopmental disorders.

### Speech

Another accessible neurophysiological function that reflects thought processing and also results from a complex integration of sensorimotor signals is represented by speech, considered in its quantitative dimensions, which has also been correlated with EEG microstate patterns [51]. Given that communication difficulties are key features of autistic and psychotic disorders [52], computational methods have recently been introduced to objectively quantify linguistic anomalies in the psychosis spectrum and to identify subtle and early linguistic peculiarities in UHR individuals [53]. Recent studies have shown that analyses in the semantic and syntactic areas could predict psychotic transition [54], but the predictive role of other linguistic domains, such as phonetics, has so far been poorly investigated. A main aspect of phonetic research is prosody, the tone of voice with which words are pronounced, crucial for communication [55]. Researchers from both the phonetic and psychiatric fields have invested significant effort into trying to precisely characterize the prosodic profile of patients with schizophrenia, generally finding reduced pitch variability and increased pause duration [56]. However, among the limitations of the existing research, are a weak generalizability of the results to languages other than English, a lack of comparisons with other clinical groups and scant attention devoted to voice quality [56]. Besides, prosodic cues have scarcely been explored in individuals with high risk of psychosis and more research is needed to clarify the potential predictive role of these features [57, 58].

Alongside traditional approaches investigating communicative behavior in psychosis focusing only on the voice of the patient, it is also necessary to investigate what happens at the interactional level [59]. Turn-taking analysis specifically explores dialogical interactional behaviors. Turn-taking is the organization of the conversation into alternating speaking turns between different interlocutors and its main goal is to assure that no more than one person is speaking at any time. Another goal is to avoid long silent gaps between the end of one speaking turn and the beginning of the next one [60]. Turn-taking analysis has rarely been applied to individuals with psychosis and at-risk mental states so far [61–63].

It appears that in this group turn-taking patterns involving increased mutual silence are prevalent. Interestingly, voice atypicalities have also been quantified in individuals with autism spectrum disorders, both in childhood and adulthood [64]. Moreover, recent studies have found an increased number of silent gaps as compared to controls in the early stages of dialogues [65, 66]. Of note, there is evidence suggesting that there are shared social cognition deficits between autism and schizophrenia spectrum disorders [67]. From this perspective, there is additional motivation for comparing prosodic and turn-taking patterns in individuals with ASD and along the psychosis spectrum.

Crucially, the possible link between prosodic and turn-taking variables and their neurophysiological substrate in microstates has never been studied in patients with these profiles.

### Self-reference effect and disorders of the self

Neurophysiological measures may also shed light on the individual’s phenomenological experience, such as self-consciousness [68], and its alteration in patients with psychotic disorders [69]. The sense of self is multifaceted and can be examined through two main prisms: firstly, as knowledge about “Me”, object of a reflexive construct of the self-concept, stored in long-term memory (narrative self) [70], and secondly as an “I” subject of the pre-reflexive and embodied subjective experience in the here and now (minimal self) [71]. Self-disorders constitute a core feature of the schizophrenia spectrum, markers of vulnerability to psychosis and predictors of psychotic conversion in patients at ultra-high risk or who had a first episode of psychosis. One of the possible prisms for studying these self-disorders is based on the evaluation of the self-reference effect on memory, according to which processing information closely related to the self is the most effective strategy for remembering new material [72]. Indeed, the self is intimately linked to memory and acts as a processing bias that determines how and what information is encoded and retrieved [73, 74], particularly in episodic memory, which refers to the memory of the past experiences of the self and contributes to one’s feeling of identity and temporal continuity. However, minimal or narrative self-disorders appear associated to an altered or even lack of self-reference effect on memory [75, 76]. Studying the self-reference effect in early psychosis could therefore contribute to characterizing the extent and course of self-disorders in prodromal (ultra-high risk) and early (first episode of psychosis) stages of schizophrenia. An innovative task has been designed using immersive virtual reality to evaluate the self-reference effect on episodic memory via a naturalistic approach, relying on the encoding of multisensory daily life events rather than simplistic lists of words or objects.

### Objectives of the DEMETER study

Building on our preliminary results, the DEMETER project (“Détermination Des Microétats EEG associés Aux Troubles Psychiques Dans Les États à Risque”—EEG Microstates Across At-Risk Mental States) is a prospective observational study that aims to characterize the EEG microstate signature with regard to underlying neurophysiological functions, including sensorimotor integration, speech, sleep, and sense of self, across a population of adolescents and young adults, with ultra-high-risk of psychosis (UHR), first-episode psychosis (FEP), schizophrenia (SCZ), autism spectrum disorder (ASD), and major depressive disorder (MDD), compared with healthy controls (CTRL) (*N* = 21 × 6), between two timepoints one year apart.

Participants will undergo deep phenotyping based on psychopathology and neuropsychological assessments at baseline and after one year of follow-up, high-resolution EEG (64 electrodes) with a resting period and a sensorimotor task, a recording of the characteristics of their speech (prosody and turn-taking), a one-night polysomnography, and biological sampling for multi-omic analyses, and a self-reference effect task in virtual reality (the latter only in UHR, FEP, and CTRL).

The main questions it aims to answer are as follows. 1) Are EEG microstate anomalies associated with specific disorders, and clinical and functional prognosis, both in resting conditions and during sleep ? 2) Are EEG microstate anomalies associated with differences in sensorimotor integration, speech, and sense of self ? 3) An interventional ancillary study will involve only healthy controls, in order to assess whether light hypnosis conditions can modify the EEG microstate architecture in a direction opposite to what is seen in disease.

## Methods

### Participant recruitment

All participants will be included at the Clinical Research Centre (CRC), University Hospital Group Paris Psychiatry and Neurosciences (GHU). Inclusion criteria are: an age between 15 and 30 years old; French as the maternal language or spoken in the context of bilingualism; a DSM-5 diagnosis of schizophrenia or major depressive disorder or autism spectrum disorder; a diagnosis of ultra-high-risk of psychosis or first-episode psychosis based on the Comprehensive Assessment of at risk mental state (CAARMS) translated in its French version [77]; and healthy control subjects recruited from the general population. Exclusion criteria are: suicidal risk; severe or non-stabilized somatic and neurological disorders; epilepsy; head trauma; IQ below 70; presence of other psychiatric disorders (bipolar disorder, obsessive–compulsive disorder, or substance use disorders, except for tobacco or cannabis, tolerated up to 5 joints/day); for healthy control subjects, a family history of psychosis is an exclusion criterion. Pregnant or breast-feeding women will not be included.

**Fig. 1:**
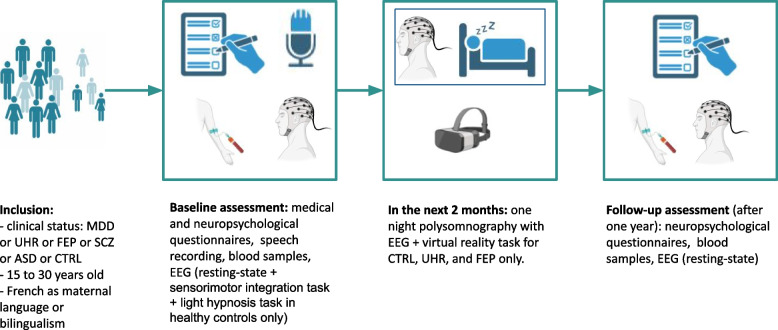
Protocol design. MDD: major depressive disorder. UHR: ultra-high-risk of psychosis. FEP: first-episode psychosis. SCZ: schizophrenia. ASD: autism spectrum disorder. CTRL: healthy controls

Participants will be screened among the population of patients seen at an early psychosis outpatient clinic (Centre d’évaluation des jeunes adultes et adolescents—CJAAD, GHU). Healthy controls will be reached through the healthy volunteers database of the CRC. Participant assessment will be as follows (Fig. 1, Table 1). 1) During the pre-inclusion visit, participants will be informed of all the details of the protocol, orally and in writing, and eligibility criteria will be verified. Then a two-week reflection period will be observed, before the signature of a written consent at the baseline inclusion. 2) The baseline visit will consist of a first visit of three half-day sessions including medical, psychopathological and neuropsychological assessments, biological sampling (described below), and speech recording. In the following two months, participants will undergo one night of polysomnography (everyone) and two half-day sessions for the sensorimotor task (everyone) followed by light hypnosis (only controls), and the self-reference effect task in virtual reality (only controls, UHR, and FEP). 3) The follow-up visit will consist of a second psychopathological and neuropsychological assessment, biological sampling, and shorter EEG recording (5–10 min).

**Table 1:**
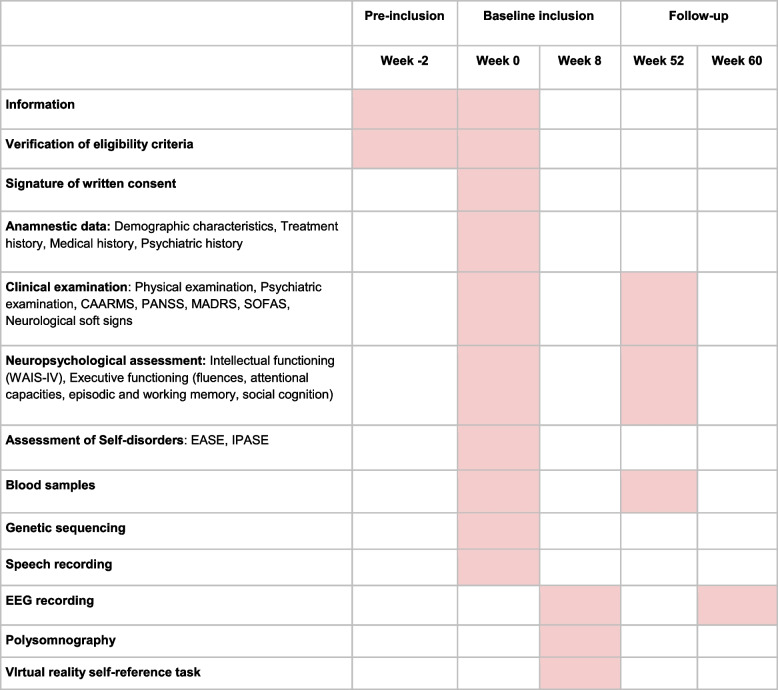
Summary and timing of assessments

*CAARMS* Comprehensive Assessment of at risk mental state, *PANSS* Positive And Negative Syndrome Scale, *MADRS* Montgomery–Åsberg Depression Rating Scale, *SOFAS* Social and Occupational Functioning Assessment Scale, *EASE* Examination of Anomalous, Self-Experience, *IPASE* Inventory of Psychotic-Like Anomalous Self-Experiences

Healthy controls will receive a compensation of 120€, and participants with a psychiatric disorder will receive a financial compensation of 60€, as they will benefit to an access to more personalized care. This protocol has been approved by the ethics committee (Comité de protection des personnes) Ouest II (approval number: 2021-A01919-32).

### Measures

#### Clinical, psychopathological and neuropsychological assessment

A clinical assessment will include anamnestic data collection (socio-demographic characteristics, treatment history, medical history, psychiatric history); a clinical examination with a physical examination, a psychiatric examination, the CAARMS, the positive and negative syndrome scale (PANSS), the Montgomery–Åsberg Depression Rating Scale (MADRS), Social and Occupational Functioning Assessment Scale (SOFAS), and neurological soft signs [78]. The neuropsychological assessment will include intellectual functioning (WAIS-IV), executive functioning (fluences, attentional capacities, episodic and working memory), and social cognition.

#### Speech assessment

Each participant will undergo a semi-structured interview with an experimenter. Interviews will focus on interests and passions, to elicit as free and spontaneous dialogues as possible. Topics related to the participants’ clinical symptomatology will not be approached, unless participants explicitly wish to do so. The recordings will not have a fixed duration, but an attempt will be made to obtain at least 15–20 min of dialogue. Both speakers will wear head-set AKG-C544L microphones, connected via AKG MPA VL phantom adaptors to a Zoom H4n Pro Handy recorder. Speech will be recorded at a sampling rating of 44,000 Hz (16-bit). The distance between the mouth and the microphone will be kept at 2 cm to ensure consistent levels of vocal loudness. Moreover, the two speakers will be placed as far as possible, to prevent crosstalk (i.e. speech of one interactant caught by the other interactant’s microphone). Finally, the recordings will be carried out in a quiet room to limit environmental noise. This is consistent with previous analyses on acoustic patterns in psychiatry [79]. The.wav files obtained will be annotated using Praat software and subsequently analyzed with Praat [80] and R. Prosodic features will be extracted using the Prosogram tool (a set of Praat scripts, open-source) [81] and voice quality features will be computed with a modified version of scripts from the Prosogram tool [82]. Turn-taking variables will be quantified with combined Praat and R scripts [62, 66, 83]. This task has been designed and will be supervised by an expert in Phonetics and by a psychiatrist trained in linguistic data extraction and analysis (MGr and VL).

### Sensorimotor integration task

Sensorimotor integration is investigated using a visuo-tactile task. On each trial, the participant, seated in front of a screen, has a visual instruction: a point to the right or left of the screen. The task consists of pressing one of the two buttons positioned on each side of the body with the index finger of the corresponding hand according to the visual instruction. A vibrotactile stimulator (small bone conduction speakers wired to an Arduino electronic card modulated by an amplifier) is applied to the first dorsal interosseous muscle of both hands. 400 msec before the visual instruction, one of the two hands receives a tactile cue (vibration) on one hand for 100 msec. The tactile cue can be congruent or incongruent with the visual cue, both indicating or not the same hand. Depending on the block, the tactile cue can be more or less reliably coupled with the visual stimulus. In *reliable* blocks, 90% of the trials present the vibration and visual instruction congruently (indicating the same hand). In *non-reliable *blocks, only 50% of the trials are congruent, and in this case, the tactile cue is not reliable. Two blocks with 70% congruent cases are carried out intermediately. Finally, a baseline block which does not contain any tactile cues is presented at the beginning and the end of the task. The order of the 90% and 50% blocks is randomized. The tactile and visual stimuli are generated with a MATLAB script. Each block consists of 100 trials, in total 500 trials. EEG data is recorded throughout the task, using a 64-channel EEG cap (from Biosemi). The setup is coupled to an eye tracker in order to control that the participant is fixing the cross at the center of the screen during each block. At the end of the task, a five minute eyes closed resting-state EEG will also be recorded. In order to examine attentional modulation, measurement of alpha power band (in Hz) is computed. Cortical excitability and inhibition are analyzed with mu and theta bands (in Hz), and integration of sensory information as somatosensory evoked potentials (SEPs), where amplitudes (in µV) and latencies (in msec) are extracted. The adaptation of the reaction time (in msec) to the button press according to the probabilistic context of congruency is examined. Analysis is conducted with Python scripts with dedicated libraries such as MNE-Python [84]. This task has been designed and will be supervised by researchers trained in neurophysiology recording and analysis (AA, LD).

### Hypnosis task

After the sensorimotor integration task, healthy controls will undergo a light hypnosis task coupled with two control tasks, in addition to the eyes-closed resting-state already recorded. First, participants will be asked to listen to a neutral text (a refrigerator manual) read by the investigator, with the instruction to listen attentively in order to be able to answer specific questions regarding the content of text. Second, participants will do a mental calculation test. Third, participants will undergo the light hypnosis task. Light hypnosis is based on Ericksonian hypnosis without inducing a trance state, and has been developed by the Collège International des Techniques par Activation de la Conscience (CITAC; Jean Becchio, Sylvain Pourchet) as part of the Paris-Saclay university training in clinical hypnosis. Participants will be asked to focus on any type of preoccupation they may have, and then to picture the first step towards resolving this preoccupation. They will then be asked to provide resources or qualities they have. The light hypnosis session will then start by asking the participant to assume a comfortable and at the same time tonic position, sitting straight, the back lifted from the chair. They will be asked to close their eyes, while being informed that they can open them at any time if needed. They will then be asked to picture their objective, and the first step toward its solution. Then, they will be asked to picture themselves in a situation where they learnt to do something. Proprioceptive, sensory (each of the five senses), and metaphorical suggestions based on their resources will be provided in order to guide the participant in this exercise. This task has been designed and will be performed by two psychiatrists trained in clinical hypnosis (AI, CLo).

### Self-reference effect task in virtual reality

Virtual reality immersion will be achieved using the HTC VIVE Pro Eye (Taoyuan City, Taiwan: HTC corporation) virtual reality headset. The self-reference effect task will consist in a walk through a virtual city, where participants will encounter a total of 32 multisensory daily life events that aim to be incidentally encoded in episodic memory. Participants will embody a virtual avatar and navigate twice through two distinct parts of the city. Prior to each navigation, avatar embodiment will be induced using a visuomotor stimulation in front of a virtual mirror, asking participants to move their different body parts while looking at them directly or in the mirror. To manipulate the minimal self-reference, one navigation will be associated with a synchronous avatar to induce a high sense of embodiment, and therefore a stronger sense of minimal self. The other navigation will be associated with an asynchronous avatar with a 700 ms-delay between participants’ real performed movements and the seen movements of the avatar, to induce a low sense of embodiment, and thus a weaker sense of minimal self. To manipulate the narrative self-reference, in each navigation path, half of the events will be associated to the participants themselves (Self), and the other to someone else (Other). The association will be induced by asking participants to take a picture of each event and rate its personal significance for either Self or Other. All conditions will be counterbalanced.

Following each navigation, participants will be submitted to self-reported questionnaires assessing their sense of embodiment (Embodiment Questionnaire) [85], sense of presence (Igroup Presence Questionnaire) [86], cybersickness (Simulator Sickness Questionnaire) [87], and current emotional state (Mood Visual Analogue Scale) [88].

Finally, participants will undergo two episodic memory tests: a free recall task and a recognition task. The free recall will be based on a verbal interview of 20 min, during which participants will be asked to recall all the events that they remember encountering in the virtual city. The recognition test will be programmed using the Python module Neuropsydia [89] and consists of displaying on a computer screen all 32 encountered events mixed with 16 lures which were not encountered in a random order, and asking participants whether they encountered this event in the virtual city. For both memory tests, participants will be asked to provide systematically and the most precisely possible, for each recalled event: description of the event, spatiotemporal situation, source, referent for the personal significance rating, perceptive and phenomenological details, degree of reliving or familiarity of the event. This task has been designed by Delphine Yeh under the supervision of Pascale Piolino, and derived from Sylvain Penaud’s protocol for the procedures linked to the minimal self-reference [90]. The virtual environment has been developed by Alexandre Gaston-Bellegarde using Unity.

### Polysomnography

An overnight polysomnography with 19 EEG channels and ventilatory polygraphy will be recorded for all participants within the first two months of inclusion, at the sleep medicine department of Hôtel-Dieu hospital, in Paris (Centre du Sommeil et de la Vigilance). Trained sleep technicians will set-up the head-sets in the evening and supervise the recording during the night. The recordings will be analyzed by trained sleep specialists (SR, LD).

### Microstates analysis

Microstate analysis will be performed on eyes-closed resting-state, during the sensorimotor integration task, and during sleep. A minimal preprocessing will be done with the MNE EEG software on Python, which includes a bandpass filter between 0.5 and 40 Hz, rereferencing to the mean, and visual and automatic correction for artifacts using independent component analysis (ICA). Each recording will be visually reanalyzed by clinical neurophysiologists to check for any residual artifact. Microstate analysis will be done using the Pycrostates package [91]. Global field power (GFP) will be determined for each participant. Only EEG topographies at GFP peaks will be retained to determine microstates’ topographies, through a modified K-means clustering. For each subject the same number of GFP peaks will be extracted and concatenated into a single data set for clustering. A combined score will be used to compute the optimal number of clusters. The resulting clusters will be backfitted to each individual maps. Temporal smoothing will be used to ensure that periods of inter-peak noise, of low GFP, did not interrupt the sequences of quasi-stable segments. For each subject, three parameters will be computed for each microstate class: frequency of occurrence (“occurrence”), temporal coverage (“coverage”) and mean duration. Occurrence is the average number of times a given microstate occurs per second. Coverage (in %) is the percentage of total analysis time spent in a given microstate. Mean duration (in ms) is the average time during which a given microstate was present in an uninterrupted manner (after temporal smoothing).

### Biological sampling

Peripheral blood samples will be collected for genetic, epigenetic, proteomic, and metabolomic studies.

### Statistical power estimates

**Fig. 2:**
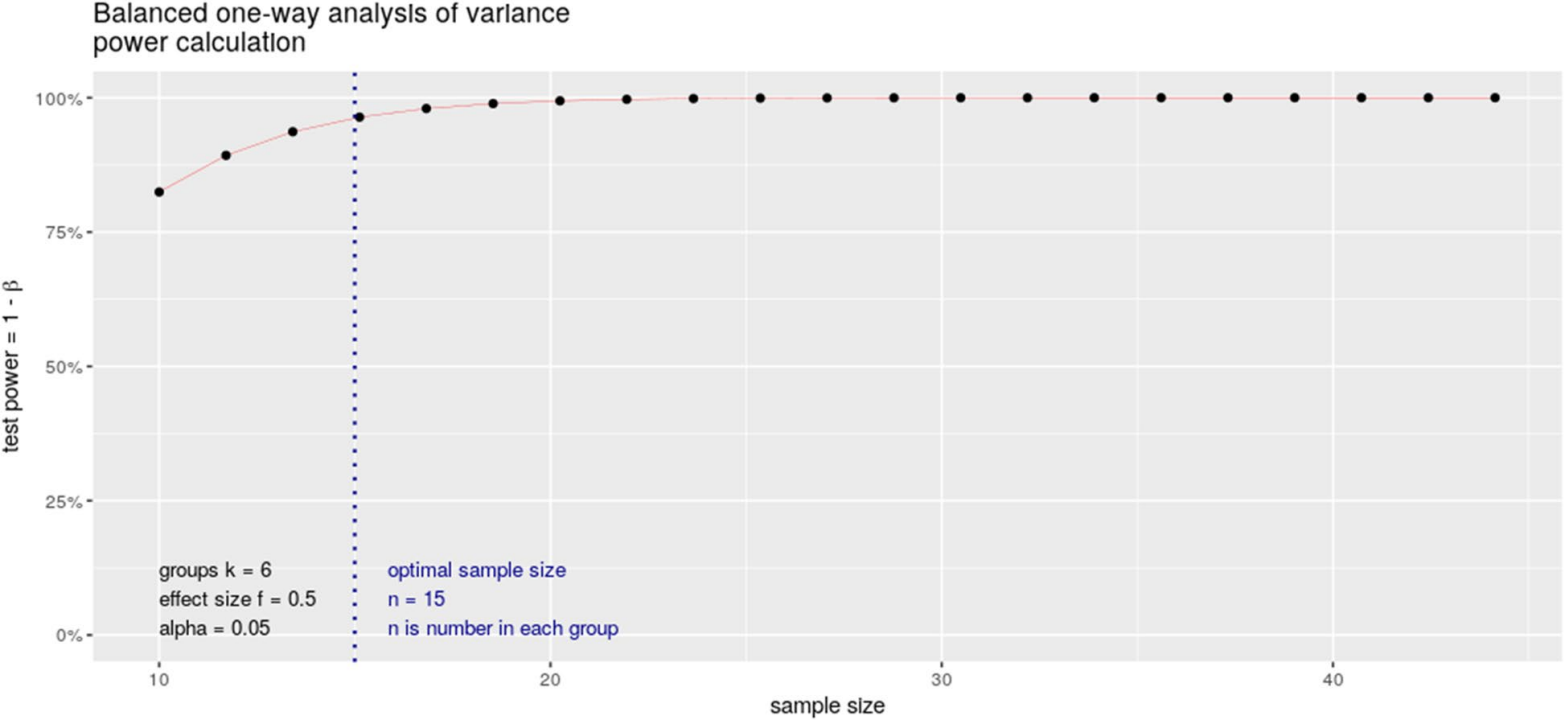
Power calculation

We considered a minimum expected effect size around 0.5, based on pairwise comparisons of EEG microstate parameters (mean duration, time coverage, occurrence) between chronic schizophrenia and relatives of subjects with schizophrenia [14]. Accepting an alpha risk of 0.05 and 95% power, we estimate the necessary number of subjects to be included in each of the 6 groups at 15 (calculated in R with the pwr.anova.test function) (Fig. 2). Given the risk of overestimating the minimum effect size associated with publications based on small cohorts, we estimate that a number of 21 subjects significantly increases the chances of obtaining a power greater than 95%. This represents a total of 126 subjects.

### Statistical analysis design

For all neurophysiological variables, the investigators will apply a repeated measures ANOVA, and use the following contrasts:

- “UHR, FEP, SCZ, ASD, MDD” vs. “Healthy subjects”, in order to test the variables as markers of general psychopathology;
- “UHR, FEP, SCZ” vs. “ASD, MDD”, in order to test the variables as specific markers of psychosis; equivalently, their specificity in MDD and ASD will be tested);
- “UHR” vs. “FEP" vs. “SCZ”, in order to test the variables as markers of state;
- Finally, in a dimensional approach, the functional correlates of microstates will be studied across wakefulness and sleep, and during speech, regardless of diagnosis.

## Discussion

The Research Domain Criteria (RDoC) strategy has led to a paradigmatic change in psychiatric research, promoting the integration of dimensional constructs beyond current nosographic boundaries. In this context, sensorimotor integration, speech, sleep–wake rhythms, and sense of self appear as relevant phenotypes to understand transdiagnostic functional impairments that lead to a high burden at the individual level [92, 93]. In a multimodal approach, the use of neurophysiological tools such as high-density EEG, polysomnography, or audio recorders offer an accessible means to study these dimensions at a very good temporal resolution. Hypnosis or virtual reality tools further give the opportunity to non-invasively modulate and test perceptions in relation with these neurophysiological assessments. Moreover, applying quantitative EEG analyses in this framework, such as microstates, may shed light on the connectivity networks underlying thought processing and provide clinically-relevant biomarkers of state that could be easily implemented in daily practice.

This protocol describes a transdiagnostic longitudinal case–control study that includes a multimodal neurophysiological assessment of sensorimotor integration, speech, sleep, and sense of self, in patients with major depressive disorder, ultra-high-risk of psychosis, first-episode psychosis, schizophrenia, and autism spectrum disorders, compared to healthy controls, in a population of adolescents and young adults aged 15 to 30 years old. Preliminary retrospective analyses from our group, with routine clinical low-resolution EEG recordings, have suggested that a variation in EEG microstates class D may be a marker of stage across psychotic disorders, as it decreases from UHR to FEP and schizophrenia. However, these changes were not specific to psychosis, and they appeared to reflect a shared dimension on the schizophrenia-autism spectrum. We also suggested that a microstate ratio imbalance between class C and class D may perhaps be more specific to schizophrenia, although it did not appear that EEG microstates were sufficient to differentiate between different groups of diseases [18]. Building on this preliminary data, we propose this prospective study with higher resolution EEG recordings to test whether anomalies in the EEG microstate architecture may be associated with diagnosis, clinical and functional prognosis, both in resting conditions and during sleep, across psychiatric disorders. We postulate that we may find EEG microstate anomalies associated with differences in sensorimotor integration, speech, sense of self, and sleep, and that the dynamic of EEG microstates may be modulated by a non-drug intervention such as light hypnosis, as a proof-of-concept of potential usefulness in psychotherapeutic approaches.

We further hypothesize that the attentional component of somatosensory integration in preparation for a motor response is modulated through visuo-tactile stimuli in healthy subjects, and is altered in patients with psychotic disorders, probably with abnormal inhibition mechanism responses. Specifically, we expect that primary sensory cortex activity, measured as alpha and beta oscillations, influences motor cortex excitability and would be desynchronized in psychosis. We also anticipate an impaired connectivity among the primary sensorimotor network, as well as altered synchrony states in an attentional context. Finally, at rest, we expect these anomalies to be associated with a C/D microstate imbalance and with microstate class E anomalies (postulated to be correlated with interoception and sensorimotor processing).

We also expect to find differences in prosodic and turn-taking patterns between patients and healthy controls. In particular, we predict all patients to display reduced pitch variability, reduced speech output and increased pause duration. Moreover, we expect patients’ voices to overlap more with the interlocutor’s. We also hypothesize that linguistic cues could be markers of the stage, with increasing levels of atypicality from UHR to SCZ. Finally, we speculate that patients with ASD and participants along the schizophrenia spectrum will present similar prosodic and turn-taking patterns.

FInally, we postulate a reduced self-reference effect on memory performance in UHR and FEP individuals, due to patients’ self disturbances. Specifically, we expect a preserved narrative self-reference effect but no minimal self-reference effect in UHR and FEP individuals, since minimal self disturbances are already present in early stages of psychosis whereas narrative disturbances of the self are less marked, as opposed to controls who are expected to exhibit both minimal and narrative self-reference effects.

This study will address several methodological challenges. Its transdiagnostic design will allow us to test the specificity of any relevant observed association. At the data collection level, the pipeline that integrates the sensorimotor task with the high-density EEG recording will follow stringent quality checks so that the EEG recordings are interpretable with regard to the underlying task. The one-night polysomnography recordings will require to anticipate all the risks associated with prolonged recordings, such as electrodes that come off during sleep due to participant movement. This implies time-consuming regular check-ups during the night from trained technicians. In order to allow reproducible results, the ancillary light hypnosis protocol will require the use of a simple standardized strategy from the two clinicians trained in hypnosis. Regarding the linguistic data collection, high-quality double-channel audio recordings are crucial to allow precise and reliable analyses with the Praat software. Regarding the self-reference effect, the innovative task using immersive virtual reality will enable to study this effect in a naturalistic and standardized context, which will capture the richness of episodic memory and its links with the self in everyday life better than the simplistic lists of words or objects that are traditionally used in self-reference effect studies. Moreover, the task will integrate both minimal and narrative self-reference, which will enable to examine under the same design the respective but also joint contributions of both facets of the self to the self-reference effect in patients with self-disorders.

In conclusion, this multimodal, transdiagnostic neurophysiological approach will help pave the way for personalized medicine through in-depth endophenotyping of sleep, speech, sensorimotor integration and self-perception, four dimensions that overlap in the spectrum of psychiatric disorders.

## Data Availability

Anonymized data will be stored at GHU Paris Psychiatrie et Neurosciences, and will be made available upon reasonable request to the authors.
